# Using Google Earth™ and Geographical Information System data as method to delineate sample domains for an urban household surveys: the case of Maroua (Far North Region-Cameroon)

**DOI:** 10.1186/s12942-019-0186-8

**Published:** 2019-11-04

**Authors:** Ronald R. B. Ngom Vougat, Steven Chouto, Sylvain Aoudou Doua, Rebecca Garabed, André Zoli Pagnah, Bernard Gonne

**Affiliations:** 1 National Advanced School of Engineering, University of Maroua, Maroua, Cameroon; 2 Department of Geography, Doctoral Research and Training Unit of “Human and Social Sciences”, University of Maroua, Maroua, Cameroon; 3 Present Address: National Institute of Cartography, Ministry of Scientific Research and Innovation, P.O. Box 157, Yaoundé, Cameroon; 4 Department of Veterinary Preventive Medicine, College of Veterinary Medicine, The Ohio State University, Columbus, OH USA; 5 School of Veterinary Medicine and Sciences, University of Ngaoundéré, Ngaoundéré, Cameroon

**Keywords:** Google Earth, Household sampling, Maroua, Cameroon, Survey

## Abstract

**Background:**

Getting a random household sample during a survey can be expensive and very difficult especially in urban area and non-specialist. This study aimed to test an alternative method using freely available aerial imagery.

**Methods:**

A gridded map and random selection method was used to select households for interviews. A hundred numbered of points were put along the edges of an updated map of Maroua. Then two numbers were randomly draw at a time and a line was drawn between those two numbers. A lot of different kinds of shapes of different sizes obtained were numbered. Ten shapes were randomly draw and the one selected were considered as ‘neighbourhoods’. A grid of 30 m × 30 m was drawn over each and then numbered. 202 grids considered here as households were randomly selected from the ten neighbourhoods for interviews.

**Results:**

Out of 202 households visited, only 4 were found to be something other than a house. In addition, 30 sampled households (14.85%) were abandoned or the occupants had relocated elsewhere. This method resulted in an accuracy level of 72%, its advantage is the ability to generate efficient random sample at relatively low cost as well the time required.

**Conclusions:**

The method proposed in this study was efficient and cost-effective when compared to the infield generation of a household inventory or Global Positioning System (GPS) tracking of households. It can then be used by researchers in low-incomes countries where funding for research is a challenge. However, this method needs to train the investigators on how to use the GPS.

## Background

As the world population continues to grow, an increasing number of people are moving into cities with the hope of securing better living conditions, good quality of education and greater economic opportunities. Globally, today, 55 percent (%) of the world’s population lives in urban areas, a proportion that is expected to increase to 68% by 2050 [1]. Urban population in Cameroon was reported at 54.94% in 2016, according to the World Bank collection of development indicators, compiled from officially recognised sources. Indeed, it appears that cities thus become the living environment of more than half of the world population.

Urban lifestyles and the increasing diversity of urban conditions have not only created new social hierarchies and cultural rules. They have also contributed to the development of a new set of roles for health care systems and the evolution of demand models for health and other resources within and between cities. Indeed for a number of reasons, city dwellers—especially those in low– and middle-income nations—are especially vulnerable to the health impacts of climate change [2]. Thus, the challenge for researchers is to analyse the complex relationships between urbanisation and health, to explore new health challenges under conditions of pervasive urbanisation, to identify universal commonalities and local specificities in the urban experience of health, in the context of globalisation and to recognise the growing interdependencies between far-flung cities [3].

A remarkable amount of research has been conducted by several disciplines, including public health, urban planning, natural sciences or epidemiology, on the potential associations between urban areas and health or well-being [4]. To do such research, it is important to make surveys.

Studying the social factors and especially those that influence the health status of the population is one of the most important issues of sociological surveys today. This state is reinforced in urban areas where it is known that, the study of spatial disparities in health is rather complex because it is influenced not only by factors which are specific to the individual but also to a whole group of others which mount in socio-economic, demographic and political environment [5]. In addition, the analysis of the impacts of environment as well as health phenomena must be done in the city because of their growing dynamics and the development that still does not follow that dynamics, expose the populations to many unexplored risks. To identify these risks, researchers need relatively reliable data collection tools which combine both cost-effective results and affordable costs. Indeed, according to Kah and Pruvot [6], one of the main sampling constraints for ‘a geographer’ is to bring out samples that are representative of the territory or society he is studying. These authors estimate that the use of original technique is necessary to reduce this gap. Because of the complexity of the survey, in many papers, the sampling methodology used is not well described [7–10].

Due to the significant population growth and subsequent occupation dynamics in most African cities, household surveys are particularly important. Indeed, household surveys are an essential option to overcome the lack of outdated, incomplete or inaccurate official data [11]. In fact, to conduct a census is quite expensive and requires a lot of time as well as the use of important human resources not always available [12]. For example, the last population census conducted in Cameroon took place in 2005 and the results were published after 5 years in 2010 [13].

Moreover, the scale of a town is rather rough if specificities and internal variations are identified. Therefore, the map is a tool of choice for sampling operations. In this order, satellite images and geographic information systems (GIS) provide many opportunities that make it possible to have a synoptic overview of the extent of a territory and an up-to-date map as well as a database. When the geographical aspect is integrated, it can be perceived that taking into account the spatial scale is very important when it comes to the study of urban behaviours related to health. Everything that would not have been possible with a simple conventional sampling method. In fact, spatial sampling is now a fundamental element of surveys in many disciplines [14]. In addition, the use of GIS today offers the possibility of having a free and easy access to geographic data. Among them, the Google Earth software makes it possible to carry out effective and inexpensive mapping approaches for developing countries [15]. The objective of this paper is to propose an alternative method of surveys which is inexpensive and simple using Google Earth and Geographical Information System data as an alternative to some outdated data.

## Methods

### Study site

**Fig. 1:**
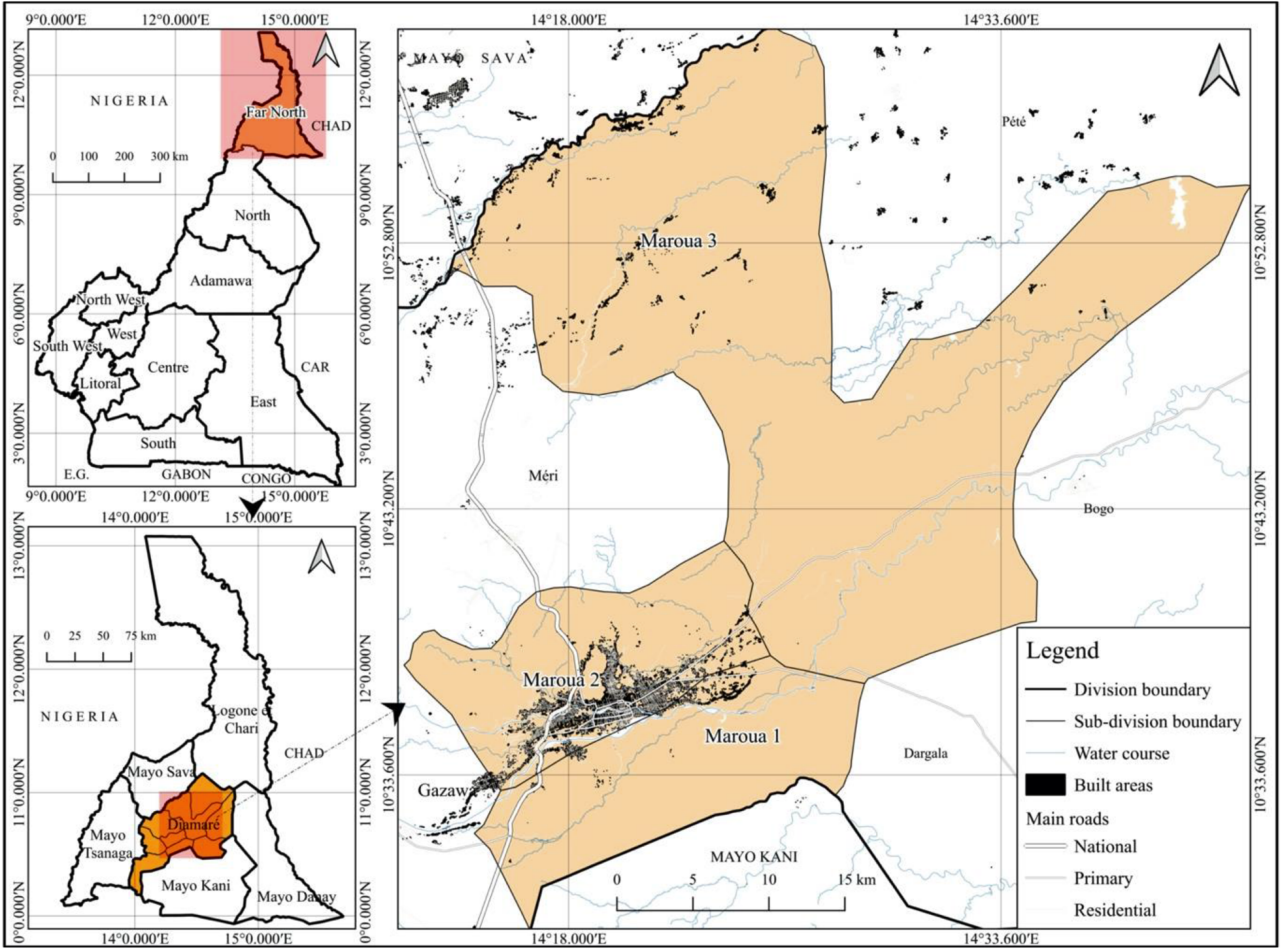
Location of Maroua in the Far North Region of Cameroon

This study was carried out from july to october 2014 in the Maroua towns which is the biggest town in the Diamare Division of the Far North Region of Cameroon. This Region is the most populated in the country after the Centre with around 3.5 million populations. Maroua is the chief-town of the Diamare Division with a surface area of 4665 km^2^ and is bounded to the South by the Mayo Kani division, to the North by Logone & Chari and Mayo Sava divisions, to the East by Mayo Danay and to the West by the Mayo Tsanaga. This Division had 784,910 inhabitants in 2013 with a density of 137.7/km^2^. The population of Maroua was 403,817 in the same year (Report of the Far North Regional Delegation of Economy, Planning and Regional Development, 2013 not published). Administratively, the city of Maroua covers three subdivisions: Maroua I, II and III. Geographically, the city of Maroua is located between latitude 10° to 13° North of the Equator and longitude 13° to 15° East of the Greenwich meridian (Fig. 1).

The main asset of the city of Maroua which justifies this study is that Maroua is a cosmopolitan city grouping together several social groups that carry out various activities such as agriculture, breeding, trade, and crafts [16]. This diversity of the population is also due to the creation of Governmental University in 2008.

Just like the Far North region, the city of Maroua is characterised by a Sudano-Sahelian climate. This climate is marked by a dry season from October to May and a short rainy season from June to September. The average annual rainfall is about 700 mm. The thermal differences are significant, with extremes temperature ranging from 27 to 41 °C. The temperatures are generally low in the rainy season and the nights in the dry season (December–January). The relative humidity of the air is quite low and varies with altitude. It varies between 30 and 35%, whereas the potential evaporation is considerable, that is from 3500 to 3700 mm [17].

### Project set-up

**Fig. 2:**
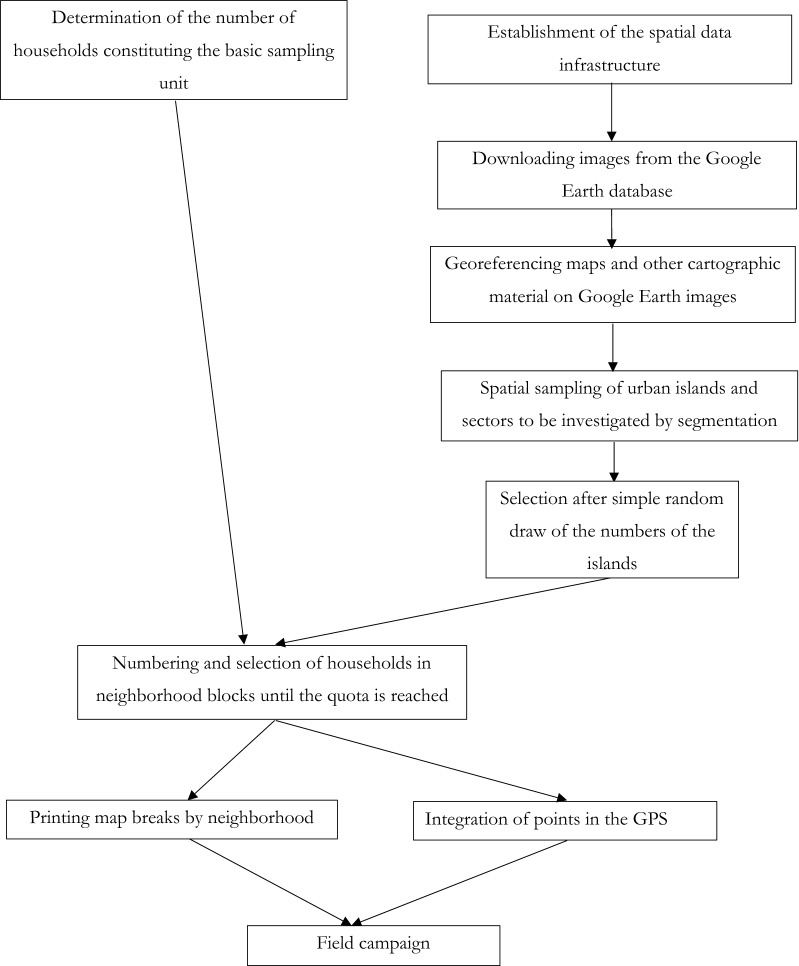
Overall methodological scheme of the study

The methodological approach adopted was to make sure to have on the same medium, a faithful overview of the spatial distribution of households of Maroua. This required the establishment of a spatial data infrastructure, the collection of images and background maps from various sources, the delineation of spatial sampling units and the design of the survey. The following flowchart (Fig. 2) shows the methodological approach described in this section.

### Establishment of the spatial data infrastructure

The establishment of the spatial data infrastructure is the prerequisite [18, 19]. The process is being conducted to coordinate and monitor the exchange and sharing of geospatial data and services. The sampling strategy used in this study was adapted from the method described by Profitós et al. [20].

### The images of the Google Earth database and other cartographic documents as data sources

To carry out this study, the Google Earth 7.1 software was used. The images from the Google Earth database are a viable alternative to provide a snapshot of the spatial pattern of a territory. The images offered by the software come from both satellites and aerial photography with a repeatability update ranging from 6 months to 5 years [15, 21]. This repetitive frequency of updating images over time makes them an effective alternative to old, non-updated data from official data collection agencies.

**Fig. 3:**
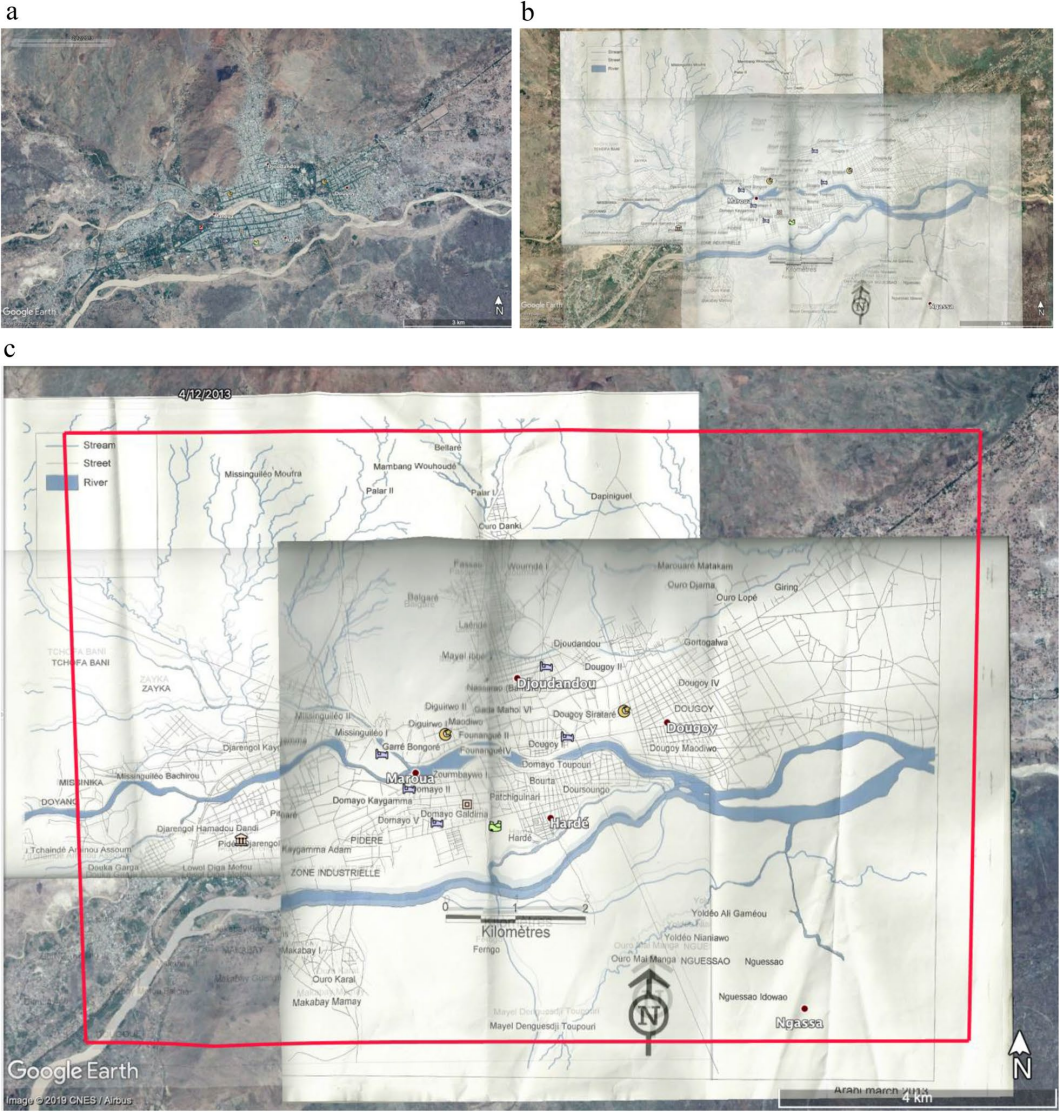
Delimitation of the study area. **a** Maroua and its peripheries; **b** scanned map of the city on satellite images; **c** delimitation of the study area

We started by updating the Maroua NC-33-6 topographic sheet at the 1/250 000 published by Army Map Service (S & H) in 1960. This topographic sheet was used as the basic cartographic infrastructure [22]. Similarly, the maps of a Non-Governmental Organisation that have worked in the city showing the location of neighbourhoods and interesting places have been used as a basis for the inventory of spatial configuration and neighbourhood distribution. To update this map, the images taken by the Digital Globe satellite on September 20, 2013 was downloaded from Google Earth 7.1 database. This operation consisted of projecting the scanned topographic sheet onto the downloaded images in order to match and georeference the immutable bitter points in the space (rivers, bridges, etc.) (Fig. 3). This process, which facilitated the delimitation of the city of Maroua, also enabled the different neighbourhoods to be identified with their names.

### Selection of the proper survey sites

#### Spatial sampling of urban islands and areas to be surveyed

**Fig. 4:**
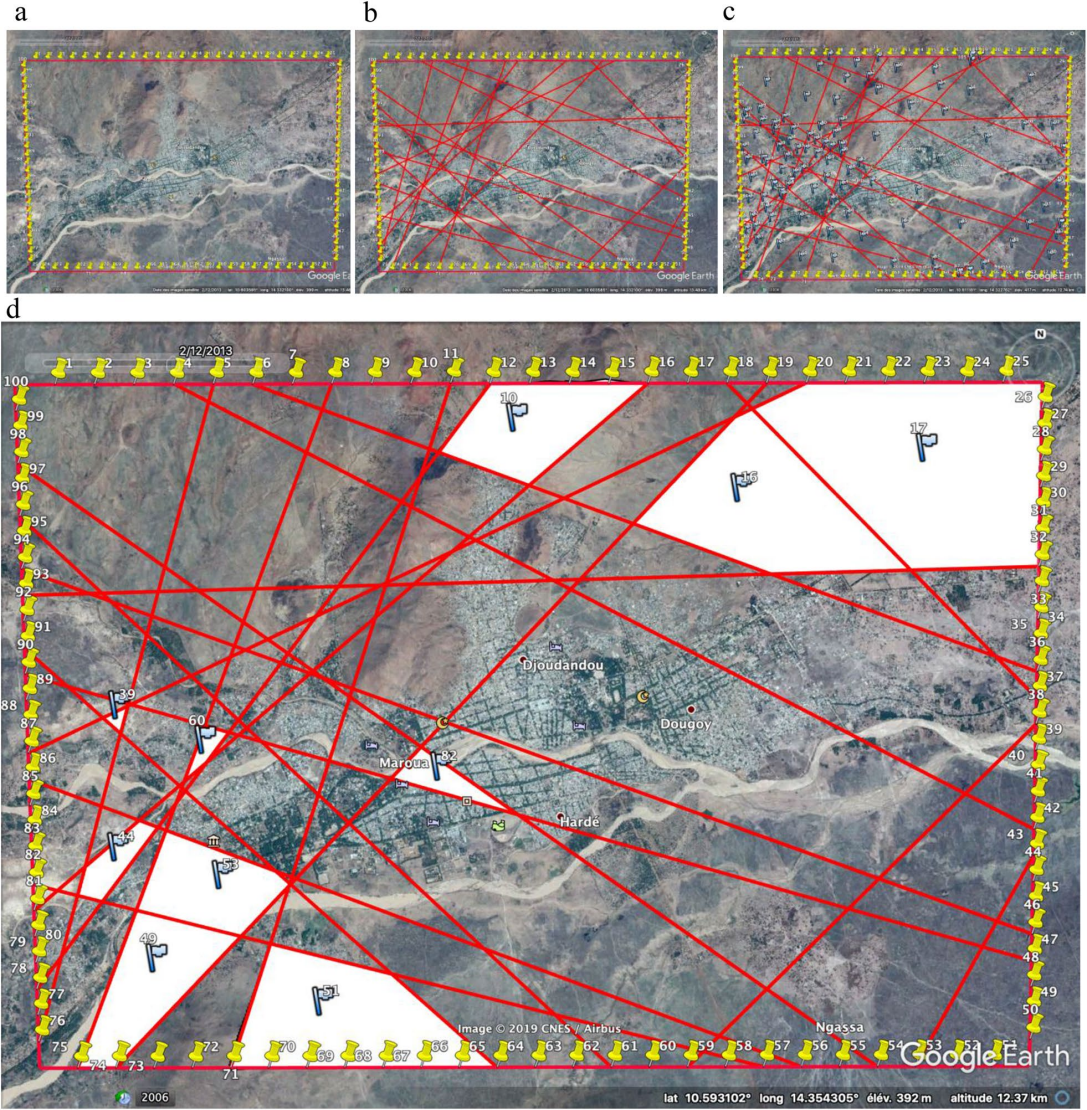
Neighbourhood delineation process. **a** Segmented border; **b** shapes of different sizes obtained after printing; **c** shapes numbered;** d** selected shapes used as ‘neighbourhoods’

After this step, 100 points were placed along the edge of the map (25 equidistant points on each side) (Fig. 4a). These points were then randomly numbered from 1 to 100.

Once the contours of the city framed and 100 points affixed, the technique used was to draw at random a couple of points. Indeed, by simple random sampling, two points on the existing 100 were drawn at the same time and a line connecting the two selected points was traced. A total of 20 pairs of points were drawn for this purpose. This allowed us to have several geometric figures of different size (Fig. 4b). All these figures were subsequently numbered randomly by a unique identifier (Fig. 4c).

#### The number of households constituting the basic sampling unit

Ten figures were then selected after a simple random draw using their numbers. These selected figures then constituted the ‘neighbourhoods’ (Fig. 4d).

**Fig. 5:**
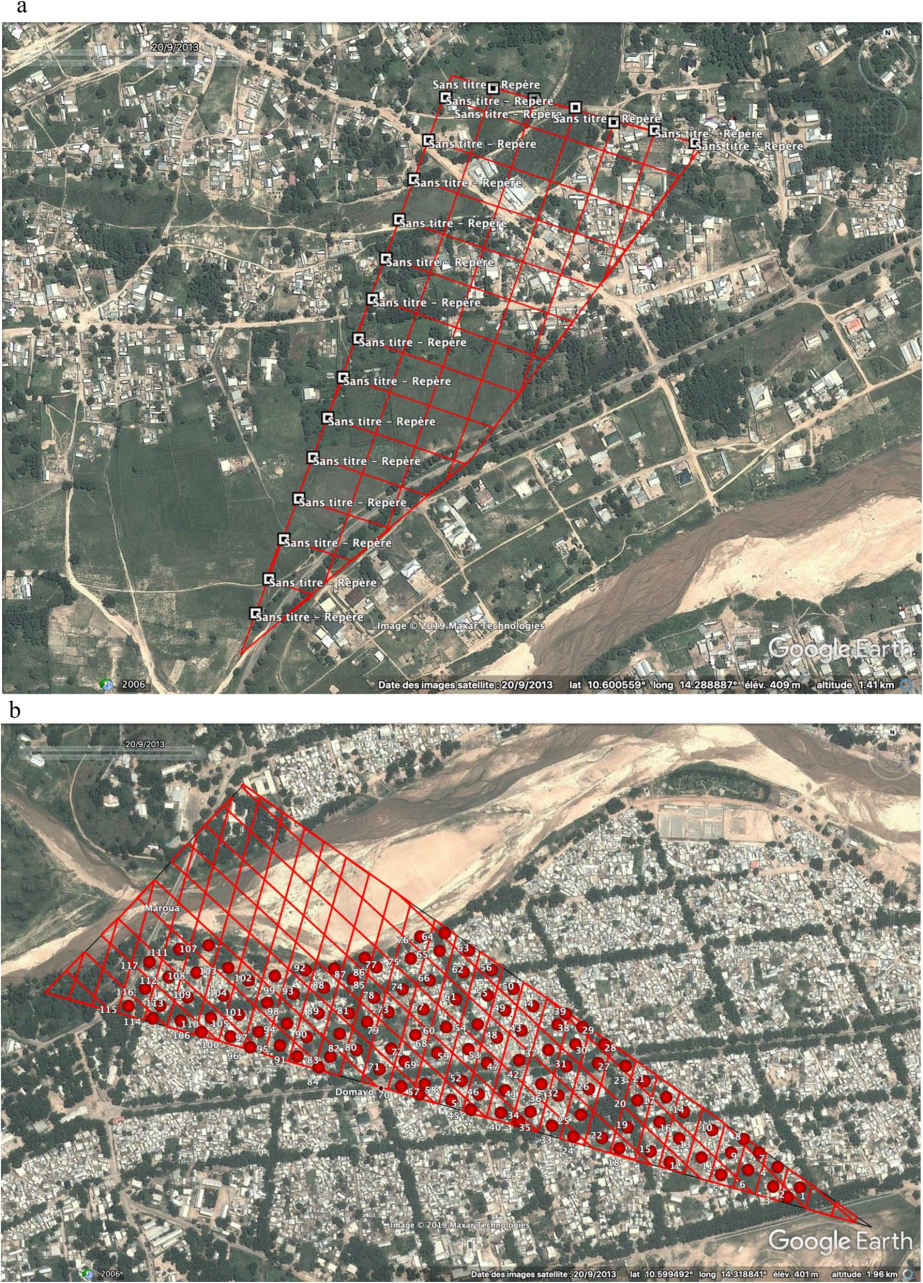
Sampling of grids and households to be surveyed. **a** Example of grids drawn in a ‘neighbourhood’; **b** numbered grids

In each ‘neighbourhood’, a grid of 30 m × 30 m was plotted as shown in Fig. 5. Indeed, according to Mayer [23] and Zhang [24], the buildings studied by remote-sensing in some urban areas in Africa have an average surface over 100 m^2^. The surface defined in this study will allow precise detection (which is close to the realities of our study area), knowing that in our study area a household can contain several concessions and this especially in polygamous households [25]. So in this context, a grid corresponds to a household. All grids obtained in each ‘neighbourhood’ were randomly numbered, except those that were located on roads, water points or bridges. Each limited urban islands have been numbered and codes were reported and processed with the software R (versions 3.1.3) [26]. Then, this resulted in a simulation of the random draws by the function ‘sample’ and thus to select the principal islands of the sector to survey. These numbers were used to randomly draw these grids (Fig. 5).

### Input data collection and preparation

Finally, in each neighbourhood, a well-defined number of grids was selected taking into account the total number of households to be surveyed (200), the total number of grids in the 10 selected neighbourhoods and the number of grids in this neighbourhood. Therefore, for a quarter name ‘A’ number of select grid was:

$$ {\text{Number of grid}}\left( {\text{A}} \right) = \frac{{{\text{Total number of grids of A}} \times 200}}{{{\text{Total number of grids of the }}10{\text{ neighborhoods}}}} $$

Once the grids were drawn, they were randomly drawn again. The coordinates of the parcels thus provided were recorded in ‘Kml’ format. Then, using the QGis version 2.6.1 software [27], the KML files were converted to ‘GPX’ file and then imported into a Garmin Etrex H branded GPS (precision 10 to 2 M, acquisition Tedm: 3 to 39 s).

## Results

### In-field assessment of accuracy of this methodology

To achieve our goal, a survey sheet with open-ended questions that focused on a number of topics was used with entries where the GPS coordinates should be noted.

On the field, the investigators were two, a man and a woman for the concerns of representativeness of the sexes. The investigators were provided with printed Google Earth images on paper indicating the sector of investigation in question. The main access routes were marked to avoid location problems. Upon arrival, the investigators had to go to the first household where the arrow of the previous steps was pointing.

In the case where the concession was empty, the investigators were instructed to go to the nearest concession adjoining the previous and located on the same axis. This procedure was also used in case of a refusal by the respondents.

Over a month, enumerators visited 202 households. Of these 202, only four were found to be something other than a house. In addition, 30 sampled households (14.85%) were abandoned or the occupants had relocated elsewhere. This method resulted in a 78.37% accuracy level.

**Fig. 6:**
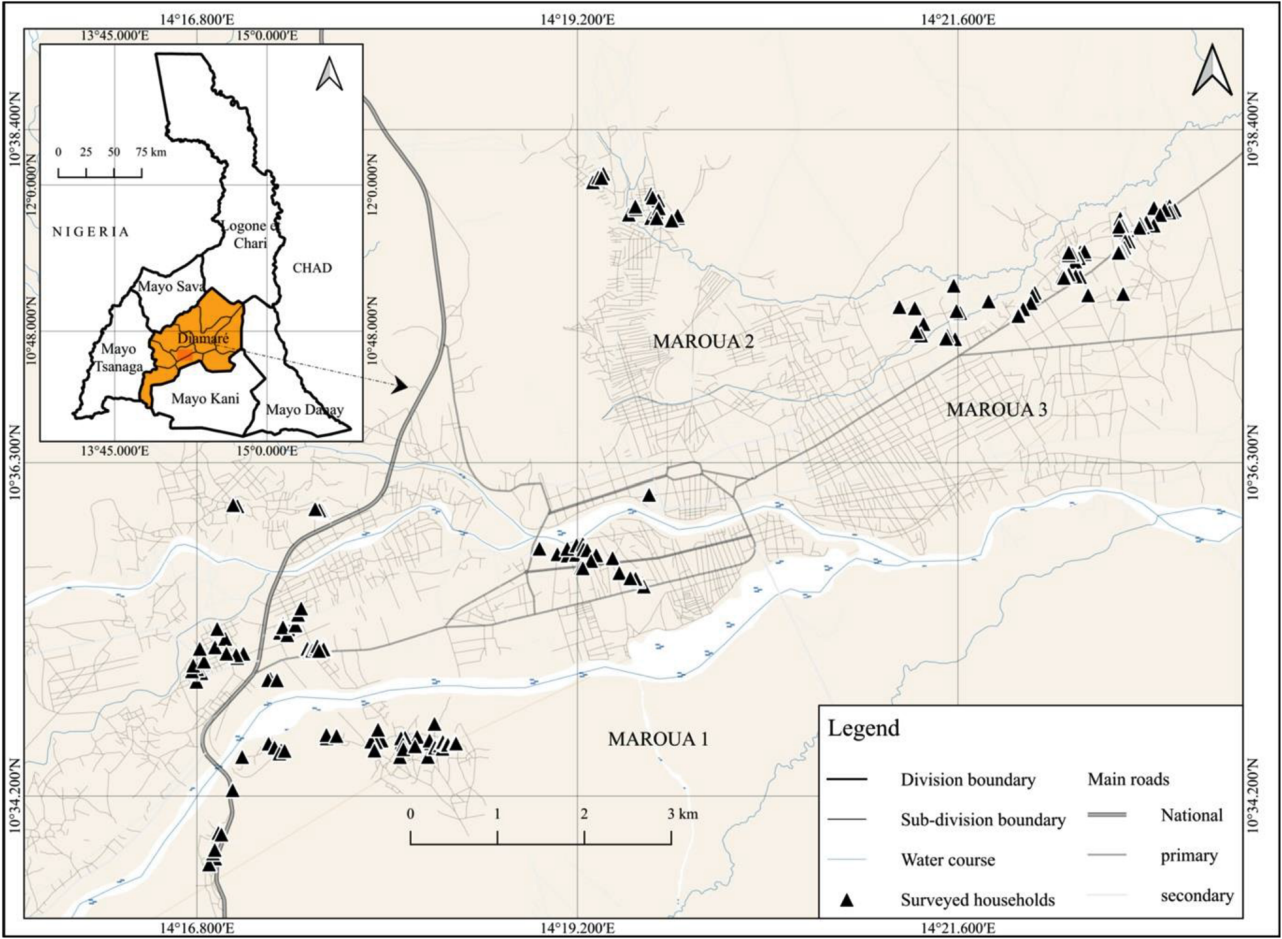
Distribution of surveyed households

Figure 6 shows the households surveyed in the urban area of Maroua.

### Distribution of surveyed households

The Tables 1 and 2 summarises the field surveys and their purpose in relation to in relation to the scheme established before the survey.

**Table 1:** Summary of field surveys

Code numbers into the Google Earth database	Neighbourhood name	Number of households surveyed	Empty house	% achieved compared to departure
10	Ouro Danki, Palar I	18	–	88.89
16	Ouro Lopé, Ouro Djama, Marouaré Mofou, Marouaré Matakam, Giring	19	5	57.89
17	Djarengol Kodek	54	–	83.33
39	Palar	2	2	0
44	Douka Garga, Douka Moussa	14	4	71.43
49	Makabay Ii, Makabay Mofou Batchar, Dougouf	18	–	94.44
53	Lowol Diga Mofou, Pirowel	23	3	73.91
51	Makabay Guisiga, Ouro Karal, Makabay Mamay	27	15	40.74
60	Missinguiléo	4	–	100
82	Domayo Galdima, Domayo I, Ii, Iv, Pont Rouge	19	1	94.74
Total		198	30	78.37 (mean)

**Table 2:** Challenges, advantages and uncertainties for each process of sampling strategy

Process	Challenge	Advantages	Uncertainties
Step 1: Delimitation of the study area	Mastery of the of Google Earth software Knowing the boundaries of the study area and the remarkable morphological features (hydrography, orography, main arteries, stadiums, etc.)	Choice of the entire study area itself upstream Is done randomly Overview of the city and all potential sampling areas regardless of their surface area	The possibility of encountering areas without habitats
Step 2: Neighbourhoods selection	Mastery of Google Earth software Delimited areas do not follow a regular grid The distances between areas are randomly selected and obey only random sampling criteria	The choice is random and easy to make with the R software Easy data cleaning process involving the previous steps Possibility to select at the finest spatial resolution Independence in the choice of sites No redundancy in the choice of neighbourhoods	Do not know in advance the number of lots/neighbourhoods that will emerge
Step 3: Household selection	Have a good internet connection speed	Precise The possibility of avoiding bias by clearly identifying habitats Advanced knowledge of the number of houses to select Random sampling and easy to make with R software	
Step 4: Integration of points in the GPS	Mastering the conversion of file formats (Kml to GPX)	Automatically done from Qgis to GPS	
Step 5: Field campaign	Training the investigator to master the use of GPS and map reading	Easy recognition on fields of previously identified sites and locations Quite inexpensive because the investigator does not waste time in identifying the field	Possibility to find new habitats set up between the date of the images and the date of the fieldwork

## Discussion

The methodology used reveals several advantages by enabling the use of combined GPS tools and the use of Google Earth™ imagery. In fact, it emerges from the survey strategy that 78.37% of the initial spatial sampling has been achieved and have been investigated as originally defined. As a result, more than a third of the sample frame was reached without any considerable effort from the investigators. At the location planned, the surveys were carried out. For the remaining 14.85%, with only 9.60% having declined to take part in the survey exercise, which are independent of the sampling plan, they come mainly from the urban dynamics of African and Cameroonian cities that are undergoing mutations and also from the respondent’s willingness to respond. However, the investigators were only required to follow the departure protocol, which was designed to help them redirect themselves to the nearest door. These statistics inform about the importance and contribution of the method used and several benefits that have been observed.

Indeed, the low percentage of no response indicates that little bias can be introduced in the information collected due to the lack of information like in previous studies [28–30]. This is a real asset in the field of health surveys, where traditional socio-demographic observation tools such as exhaustive censuses and probability sample surveys are difficult to be implemented and do not adequately meet the needs of research and urban planning [31].

Another strength of our approach comes from the fact that it saves time and make it easier to manage time effectively. The procedure for designating the neighbourhood and house to be investigated is done before surveys. As in analogous studies [14, 28, 32], the time taken to prepare the sample prior to the field trips guarantee a speed of execution once it has been implemented. In epidemic areas, this methodology appears to be ideal in the situation where there are no survey databases [33–35]. Indeed, the cost of data collection and the length of time required to conduct traditional censuses limit the frequency of surveys to about 10 years, which is insufficient for monitoring rapidly growing urban populations or health issue [31]. This would compromise any chance of implementing solutions to urgent health problems. This is a significant gain in time for the continuity of investigation and its implementation by field investigators.

In addition, the survey manager, with the use of GPS and printed maps, has an overview of the evolution of data collection in the field and can if necessary change the sampling design when he when ever need arises. This combination of GPS receiver and printed maps allow to overcome common measurement errors in previous studies [28, 36–38].

The planner has the opportunity to know if his sampling plan is followed. This is a significant contribution in the control and exploitation of data [32]. This also clearly demonstrates the reliability of the data collected in this study. Throughout the survey progress, the survey planner gathers the information collected in the field and can see the evolution of the surveys at the end of each day. As a result, the data collected is more reliable because an overview and a visual validation of the progress of the surveys can be made.

As Shannon et al. [36] stated that, the images from the Google Earth database proved accurate and with a spatial resolution that allowed the interviewers to find themselves quite easily indeed, the scenes were taken from the images of the Digital Globe constellation that offers a metric resolution. Pearson et al. [39] and Lin and Kuwayama [40] have also corroborated this state in their random surveys where the method used was also quite inexpensive. In fact, traditional methods of spatial data collection require heavy investments for census mappings prior to any intervention on the ground. Thus, it avoids repeated round trips in the field before the real survey and also avoid an increase in the budget by the recruitment of people specialised in mapping as well as lead surveyors.

However, this method nevertheless has some limitations. It should be noted that the images from the Google Earth database, although they provide a very accurate overview of the spatial configuration and distribution of households, it only give a view to a specific date. Indeed, the time of available image updates and the start of the surveys can affect the conduct of field investigations. During the fieldwork, the percentage of the habitats that were supposed to be households were found not occupied. This is due to the fact that the city of Maroua is a city with a hight rate of urban dynamic and development. The process of urbanisation has been accelerated in recent years and large parts of the city are filling up rapidly [8]. However, the images acquired from the Google Earth database have an update period. Similarly, between acquisition, printing and fieldwork, a certain amount of time is consumed.

It is also important to mention that the procedure prior to the field trip, from the setting up of the spatial data infrastructure to the selection of survey locations, is done manually. This implies that the survey planner must have a fairly reliable knowledge of the locations where the survey will be planned. Similarly, these preliminary procedures require time for the preparation of the projected maps. However, this time is quickly recovered with the reliability and speed on field trips.

In order to use this method efficiently the investigators most be drilled on data collection technique using field tools like the GPS. In fact, the investigators were trained to read the maps and to identify the different landmarks in the field. Similarly, they were trained to find previously saved waypoints. In the field, the investigators sometimes ended up in unoccupied habitats or households that refused to give answers to their questionnaires. In this case, the investigators were recommended to adapt the sampling plan by accessing the nearest household. This approach was a challenge for the investigators at the beginning in the sense that they had to judge by themselves and choose the household to be surveyed. Nevertheless, this work is a step in the direction of the reduction of uncertainty encountered in spatial analysis particularly in geography and GIS science [30].

On the other hand, it appears that this method can be even more valued for repeated demographic surveys. In fact, in addition to the ease with which the method can be approached more easily by surveys, this method makes it possible to overcome the time constraint that affects single-pass surveys. Similarly, with a georeferenced database, investigators have accurate spatial coordinates of their sample and can more easily intervene in the field.

## Conclusion

In developing countries, spatial sampling is subject to many challenges. Researchers need reliable data collection tools that combine both cost-effective results and affordable costs. Therefore, in this article, we have proposed an alternative method, which is both cost-effective and easy to be implement, and which could be an alternative to some outdated data when compared to the infield generation of a household inventory or GPS tracking of households. However, this method needs to train the investigators on how to use the GPS. The methodology shows the contribution of free Geographical Information Systems in data collection, which is intended to be quite representative for the city of Maroua. With a data collection accuracy of more than 78% of the starting quota that was found to be interested in more than one way. The need to study social factors in particular those that influence the health status of the population is nowadays one of the most important issues in sociological surveys, so they need to have a spatial sampling frame at a lower cost. All these shows that this methodology can really serve as a decision-making tool in developing countries and for low-cost research.

## Data Availability

The datasets used/or analysed during the current study are available from the corresponding author on a reasonable request.

## Abbreviations

GPS: Global Positioning System
GIS: geographic information system
°C: degrees Celsius
